# A Rare Case of Gastric Volvulus Post Laparoscopic Gastric Greater Curvature Plication

**DOI:** 10.1055/s-0040-1701224

**Published:** 2020-02-11

**Authors:** Antoine Kachi, Khalil Chidiac, Charif Khaled

**Affiliations:** 1 Department of General Surgery, Faculty of Medical Sciences, Lebanese University, Beirut, Lebanon; 2 Department of General Surgery, Lebanese Hospital Geitaoui University Medical Center, Beirut, Lebanon; 3 Department of Gastroenterology, Lebanese Hospital Geitaoui University Medical Center, Beirut, Lebanon; 4 Senior Resident, Department of General Surgery, Faculty of Medical Sciences, Lebanese University, Beirut, Lebanon

**Keywords:** gastric volvulus, laparoscopic gastric greater curvature plication, bariatric surgery

## Abstract

Gastric volvulus is a rare entity. Its diagnosis remains tricky and challenging. In recent years, the incidence of gastric volvulus has shown a rise in postbariatric surgery patient. Several cases were reported of gastric remnant volvulus post-laparoscopic sleeve gastrectomy and laparoscopic gastric bypass. Laparoscopic gastric greater curvature plication is a new and experimental restrictive technique for weight loss. Several of its complications were reported in the literature but never was a case of volvulus postgastric plication reported, as far as we know. We present this rare case with an atypical presentation and go through similar cases in the literature.

This article presents a rare case of gastric volvulus in a patient with a surgical history that includes laparoscopic gastric greater curvature plication (LGGCP). The article tackles how the case was managed and conducts a literature review of similar cases, but post laparoscopic sleeve gastrectomy (LSG) and laparoscopic gastric bypass.

## Case Presentation

We present to you the case of a 24-year-old healthy female lady with a body mass index (BMI) of 27. Her medical history includes morbid obesity (history of BMI of 41) treated by LGGCP at another institution, 5 years prior to presentation (PTP). She also suffered from upper gastrointestinal (GI) bleed that was treated by endoscopic clipping 2 years PTP. Two months PTP, she had idiopathic acute pancreatitis.

She presented to Lebanese Hospital Geitaoui—University Medical Center, for severe epigastric pain and postprandial vomiting for 3 days. Her vitals showed mild tachycardia at 110 bpm. Her physical exam was significant for severe epigastric tenderness. Labs performed 3 days PTP showed an amylase level six times higher than the normal (790 U/L) and a lipase level 1.5 times higher than the normal (230 U/L). Labs upon admission showed hemoglobin of 10.6 g/dL, white blood cell count of 13,300/mm ^3^ , but normal levels of pancreatic enzymes.

She was admitted under the assumption of chronic pancreatitis/pseudocyst. She was started on supportive treatment.

**Fig. 1:**
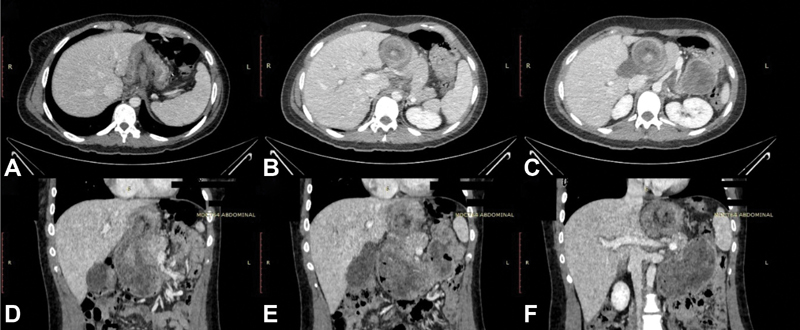
CT scanner of the abdomen and pelvis with intravenous contrast showing abnormal displacement of the entire stomach to posterior of the SMA and SMV. The antrum is shown on the left side consistent with mesenteroaxial gastric volvulus. The GEJ is shown inferior to the antrum and the pancreas has an inverted **V** -shape. ( **A–C** ) Axial reformat. ( **D,E** ) Sagittal reformat. CT, computed tomography; GEJ, gastroesophageal junction.

Nevertheless, the patient kept deteriorating within the next 24 hours. CT scan of the abdomen and pelvis was done ( Fig. 1 ). It showed an abnormal displacement of the entire stomach to the posterior of the superior mesenteric artery (SMA) and superior mesenteric vein (SMV) with the antrum on the left side, consistent with mesenteroaxial gastric volvulus. The gastroesophageal junction (GEJ) was seen inferior to the antrum. Also noted was the pancreas having an inverted **V** -shape.

The diagnosis of gastric volvulus was established. Urgent gastroscopy was attempted but failed due to the impossibility to pass the GEJ because of an obstruction at that level.

The patient was informed of her situation and signed consent was obtained for an urgent operation for reduction of the volvulus. The anesthesiologist cleared her for an urgent surgery and classified her as American Society of Anesthesiologists class II.

**Fig. 2:**
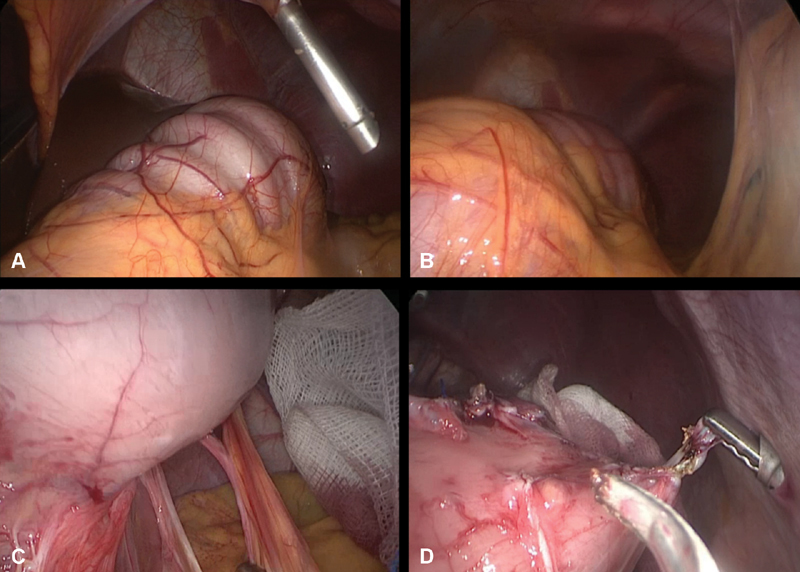
Intraoperative laparoscopic view. ( **A,B** ) Spastic and distended stomach with 90 degrees (mixture of volvulus) rotation around itself toward the liver. ( **C** ) Dissection of adhesions to the stomach. ( **D** ) Removal of all suture remnants from the antrum and up to the fundus.

The patient was transferred to the operating room and put in a modified French position-tilted reverse Trendelenburg. A 11-mm trocar was inserted via an open laparoscopic technique through a 1-cm incision 5 fingerbreadths below the xiphoid process. Insufflation of the abdomen was achieved with CO _2_ at a pressure of 12 mm Hg. Then, a 10-mm 30-degree lens was inserted and we identified a spastic and distended stomach with 90-degrees (mixture of volvulus) rotation around itself toward the liver. Three other trocars were inserted as follows: 5-mm subxiphoid used for liver retraction, two 5-mm right and left paraumbilical trocars used to insert working instruments, monopolar cautery, and vessel sealing device, as needed. Several adhesive bands were seen and liberated using a 5-mm LigaSure. Dissection was performed through the fibrotic line and into the gastro-gastric space using monopolar cautery fixed on an L-hook and scissors. As the dissection was continued, we identified two layers of suture lines. Removal of all suture remnants was done, starting at the antrum and reaching the fundus ( Fig. 2 ). Finally, the plication was fully reversed and the stomach was brought back to its original shape. Hemostasis was secured and the abdomen was desufflated.

**Fig. 3:**
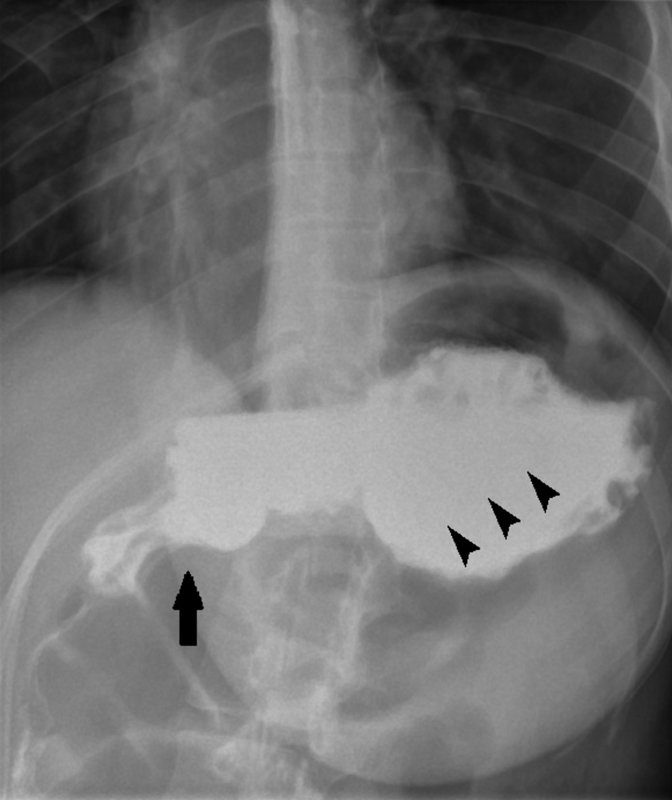
Upper gastrointestinal series. Image shows a normal-shaped stomach ( *arrowheads* ) and a good passage of water-soluble oral contrast into the duodenum ( *short arrow* ). Absence of any leakage or stenosis.

The operation took around 2 hours and went smoothly with minimal blood loss. The patient was transferred postoperatively (post-op) to the surgery ward. She was kept fasted for the following 24 hours. Day 2 post-op, an upper GI series was done and showed a normal stomach with no leaks ( Fig. 3 ). Day 3 post-op, the patient had a full recovery and she was discharged home.

Follow-up visits were unremarkable and the patient reported no symptoms of any kind. Gastroscopy was repeated 5 months later and was normal.

## Discussion

LGGCP is a strictly restrictive bariatric surgery procedure. 1 It is done by first liberation of gastrocolic, gastrosplenic, and gastrophrenic ligaments. Then division of the posterior gastric attachments is achieved. After that, invagination and plication of the gastric greater curvature are done using a 2–0 nonabsorbable sutures, over a Faucher tube ranging in size from 32 to 38 F. 1 2 3 4 So this is a reversible technique that results in the reduction of the volume of the stomach without tissue resection. 1 Nevertheless, this technique remains experimental and the American society for metabolic and bariatric surgery published that plication procedures should be considered investigational and performed under a study protocol. 5

Several articles were published detailing the post-op complications of this type of surgery. Andraos et al 2 reported obstruction due to intraesophageal fold invagination. They also reported gastric fold complications like gastric fold edema, rupture, and herniation. Skrekas et al 1 and Khidir et al 3 reported several cases of severe nausea and vomiting requiring readmission but which resolved with only supportive treatment. Nevertheless, there have been no reported cases of gastric volvulus post-LGGCP as far as we know. We found several case reports of gastric volvulus post-LSG and even volvulus of the stomach remnant post-gastric bypass. One case was even reported post-removal of a gastric band. 6 To note most of the reported cases were associated with diaphragmatic eventration.

As with most bariatric surgeries, the newly formed stomach pouch is left free from most of its attachment, which puts it at a higher risk of volvulus. Gastric volvulus or torsion is defined as an abnormal rotation and overstretching of the stomach. It can occur along the mesenteroaxial plane of the stomach (short vertical axis perpendicular to the cardiopyloric line), along the organoaxial plane (long vertical axis between the pylorus and the cardia, most common) or both. 7 It is a rare pathology and only a few hundred cases have been reported in the literature. 8

Gastric volvulus has been divided into two categories, primary subdiaphragmatic and secondary supradiaphragmatic. The primary subtype occurs due to adhesions, tumors, or laxity/absence of gastric ligaments. The secondary type is related to diaphragmatic pathology and patients in this group develop paraesophageal hernia which leads to volvulus. 9

The extensive liberation of gastric ligaments puts it at risk of volvulus. This risk increases especially after liberation of the posterior gastric attachments. Additional risk factors include incorrect positioning of the stomach postsurgical manipulation and asymmetrical plication or sleeve changing the long axis of the stomach. 10 In summary, any surgery changing the anatomy, positioning, or attachments of the stomach, puts the patient at risk of developing gastric volvulus in the future. This risk rises if the patient suffers from any diaphragmatic abnormalities.

Volvulus presents acutely as a GI obstruction. Symptoms include abdominal distention and severe abdominal pain. 7 Classic triad is the Borchardt's triad which consists of retching without any vomiting, severe epigastric pain, and inability to pass nasogastric tube into the stomach. 11 Volvulus can be complicated by ischemia and can lead to gastric necrosis, shock, and even death if left untreated.

Diagnosis of gastric volvulus is usually tricky as most physicians do not suspect it early. Murcia et al 12 found endoscopy very valuable for the diagnosis of gastric volvulus post-LSG. Nevertheless, CT remains the most useful tool for diagnosis. Sinwar 8 used CT and upper GI series. Light et al 13 described the advantages of CT as consisting of 24-hour access, speed, and assessment of gastric viability. They reported a 100% sensitivity. A study conducted by Verde et al 14 also reported that multidetector CT allows for rapid characterization of volvulus and its complications. Oral contrast, if needed to properly distend the stomach and evaluate the gastric wall, should restrain to water. Verde et al 14 also found CT helpful for diagnosing the conditions predisposing the volvulus.

Treatment of gastric volvulus is most often surgical. Various techniques have been tried, from nasogastric tube decompression to endoscopy ± stent placement. Light et al 13 have even described conservative management. Nevertheless, surgery remains the gold standard in most cases. Moreover, conservative and endoscopic treatments have a high risk of recurrence.

Various procedures have been suggested and described: gastric detorsion + lysis of adhesions, 12 gastric detorsion + lysis of adhesions + gastropexy, 12 antrectomy with gastroenteric anastomosis, 10 shortening of the lesser omentum, 7 and conversion to Roux-En-Y gastric bypass in the presence of gastric obstruction and/or gastric necrosis. 6 15 In the setting of LGGCP, deplication and removal of the sutures ± gastropexy seems to be an effective and safe means of treatment. This could be followed in a few months by LSG for weight loss if the patient wished or needed so.

## Conclusion

Bariatric surgeries impose a risk of gastric volvulus due to the extensive liberation of the attachments of the stomach. This diagnosis should be kept in mind when dealing with an acute abdomen with vomiting in a patient operated by a weight loss surgery.

Moreover, despite being introduced as an alternative to LSG, LGGCP is still an experimental bariatric surgery. It should only be done on a trial basis and by expert laparoscopic surgeons. If the sutures and invagination are not aligned properly, this would lead to a change in the long axis of the stomach and further increase the risk of volvulus.

## References

[1] Skrekas G Antiochos K Stafyla V K Laparoscopic gastric greater curvature plication: results and complications in a series of 135 patients Obes Surg 2011 21 11 1657 1663 21898042 10.1007/s11695-011-0499-6

[2] Andraos Y Ziade D Achcouty R Awad M Early complications of 120 laparoscopic greater curvature plication procedures Bariatric Times. 2011 8 09 10 15

[3] Khidir N Al Dhaheri M El Ansari W Al Kuwari M Sargsyan D Bashah M Outcomes of laparoscopic gastric greater curvature plication in morbidly obese patients J Obes 2017 2017(7989714):7.989714E6 10.1155/2017/7989714 PMC5576404 28900545

[4] Zerrweck C Rodríguez J G Aramburo E Revisional surgery following laparoscopic gastric plication Obes Surg 2017 27 01 38 43 27220850 10.1007/s11695-016-2242-9

[5] Brethauer S; Clinical Issues Committee.ASMBS policy statement on gastric plication Surg Obes Relat Dis 2011 7 03 262 21621164 10.1016/j.soard.2011.03.004

[6] Pirmadjid N Pournaras D J Huan S Sujendran V Mesentero-axial gastric volvulus after removal of laparoscopic adjustable gastric band Ann R Coll Surg Engl 2017 99 02 e58 e59 27791422 10.1308/rcsann.2016.0313 PMC5392836

[7] Ajao O G Gastric volvulus: a case report and a review of literature J Natl Med Assoc 1980 72 05 520 522 7381959 PMC2552446

[8] Sinwar P D Gastric mesenteroaxial volvulus with partial eventration of left hemidiaphragm: a rare case report Int J Surg Case Rep 2015 9 51 53 25723749 10.1016/j.ijscr.2015.02.034 PMC4392337

[9] McElreath D P Olden K W Aduli F Hiccups: a subtle sign in the clinical diagnosis of gastric volvulus and a review of the literature Dig Dis Sci 2008 53 11 3033 3036 18465247 10.1007/s10620-008-0258-2

[10] Del Castillo Déjardin D Sabench Pereferrer F Hernàndez Gonzàlez M Blanco Blasco S Cabrera Vilanova A Gastric volvulus after sleeve gastrectomy for morbid obesity Surgery 2013 153 03 431 433 22316437 10.1016/j.surg.2011.12.023

[11] Borchardt M On the pathology and therapy of gastric volvulus [in German] Arch Klin Chir 1904 74 243

[12] Murcia C Quintero P Rabaza J Gonzalez A Laparoscopic management of gastric torsion after sleeve gastrectomy CRSLS 2015 19 01 1 4. Doi: 10.4293/CRSLS.2014.00143

[13] Light D Links D Griffin M The threatened stomach: management of the acute gastric volvulus Surg Endosc 2016 30 05 1847 1852 26275540 10.1007/s00464-015-4425-1

[14] Verde F Hawasli H Johnson P T Fishman E K Gastric volvulus: unraveling the diagnosis with MPRs Emerg Radiol 2019 26 02 221 225 30644001 10.1007/s10140-019-01669-0

[15] Subhas G Gupta A Sabir M Mittal V K Gastric remnant twist in the immediate post-operative period following laparoscopic sleeve gastrectomy World J Gastrointest Surg 2015 7 11 345 348 26649158 10.4240/wjgs.v7.i11.345 PMC4663389

